# Orthodontic Traction of a Deeply Impacted Mandibular Second Molar With Severe Root Dilaceration in Close Proximity to the Inferior Alveolar Canal: A Case Report

**DOI:** 10.1155/crid/2855397

**Published:** 2026-09-07

**Authors:** Mutaz Arman, George Dahabreh

**Affiliations:** ^1^ Department of Orthodontics, Faculty of Dentistry, Al Quds University, Jerusalem, State of Palestine, alquds.edu

**Keywords:** case report, impacted mandibular second molar, orthodontic traction, root dilaceration, temporary anchorage devices (TADs)

## Abstract

**Background:**

Impaction of mandibular second molars is uncommon, particularly when associated with severe root dilaceration and intimate proximity to the inferior alveolar canal. Such cases present considerable diagnostic and therapeutic challenges because surgical extraction may result in neurosensory injury. This case report describes the successful interdisciplinary management of a deeply impacted mandibular second molar using controlled orthodontic traction supported by skeletal anchorage.

**Case Presentation:**

An 18‐year‐old female presented seeking orthodontic treatment because of dissatisfaction with her smile esthetics. Clinical examination demonstrated a skeletal Class I relationship with increased vertical facial proportions, mild mandibular deviation, posterior crossbite, and reduced overjet and overbite. Panoramic radiography and cone‐beam computed tomography (CBCT) revealed a deeply impacted mandibular left second molar with severe root dilaceration, intimate proximity to the inferior alveolar canal, and a pericoronal radiolucency suggestive of a dentigerous cyst.

Following interdisciplinary consultation with oral and maxillofacial surgeons, orthodontic traction was selected as a conservative alternative to surgical extraction. Initial treatment included maxillary expansion, alignment, and space creation, followed by surgical exposure, marsupialization of the pericoronal lesion, and attachment of a gold chain. Controlled orthodontic traction was subsequently performed using fixed orthodontic appliances, temporary anchorage devices (TADs), and a customized auxiliary levering arm.

**Results:**

After 30 months of active treatment, the impacted mandibular second molar erupted successfully into functional occlusion while maintaining pulp vitality and without clinical evidence of inferior alveolar nerve injury. A stable occlusion was maintained throughout the 6‐month follow‐up period.

**Conclusion:**

This case demonstrates that carefully planned interdisciplinary management combined with controlled skeletal anchorage‐supported orthodontic traction can provide a safe and effective alternative to surgical extraction in selected patients with deeply impacted mandibular second molars presenting with severe root dilaceration and close proximity to the inferior alveolar canal.

**Clinical Take-Home Message**

Deep impaction, severe root dilaceration, and close proximity to the inferior alveolar canal should not be considered absolute contraindications to orthodontic treatment. Careful CBCT‐based diagnosis, interdisciplinary treatment planning, and well‐controlled biomechanics using skeletal anchorage may allow successful preservation of the tooth while avoiding the potential neurosensory complications associated with surgical extraction.

## 1. Introduction

The eruption of permanent teeth is a complex biological process regulated by coordinated interactions among genetic, molecular, and environmental factors. Disturbance of this process may result in tooth impaction, a clinically significant condition that may compromise occlusal function, periodontal health, and facial esthetics [1, 2].

Although third molars represent the most frequently impacted teeth, impaction of permanent mandibular second molars is relatively uncommon, with a reported prevalence ranging from 0.06% to 3% [3–6]. The etiology is multifactorial and includes space deficiency, ectopic positioning of the tooth germ, mechanical obstruction, an abnormal eruption path, ankylosis, and the influence of adjacent anatomical structures [4–9].

Deeply impacted mandibular second molars present unique therapeutic challenges because prolonged eruption disturbance may alter root development and result in severe root dilaceration. Furthermore, dentigerous cysts may contribute to both tooth displacement and abnormal root morphology by disrupting Hertwig′s epithelial root sheath during root formation [10].

Management remains controversial and may include observation, surgical extraction, coronectomy, spontaneous eruption following removal of the third molar, or orthodontic uprighting and traction [7–9, 11–17]. However, when the impacted tooth lies in intimate proximity to the inferior alveolar canal, surgical intervention carries a significant risk of temporary or permanent neurosensory injury, making conservative treatment strategies particularly attractive.

Although several reports have described orthodontic uprighting of impacted mandibular second molars, successful management of a deeply impacted mandibular second molar presenting simultaneously with severe root dilaceration, horizontal orientation, and intimate proximity to the inferior alveolar canal remains rarely reported in the literature [12–18].

Therefore, the aim of this case report is to describe the successful interdisciplinary management of such a case using CBCT‐guided diagnosis, temporary anchorage devices (TADs), and individualized orthodontic biomechanics.

## 2. Case Presentation

### 2.1. Patient Information

An 18‐year‐old female presented to the Department of Orthodontics, Faculty of Dentistry, Al‐Quds University, seeking orthodontic treatment because of dissatisfaction with her smile esthetics. She reported no pain, swelling, or sensory disturbances in the left posterior mandibular region. Her chief concern was dissatisfaction with her smile esthetics and the alignment of her teeth.

The patient was medically fit and had no history of systemic disease, previous hospitalization, or long‐term medication use. She reported no known drug allergies and no family history of dental eruption disturbances, craniofacial anomalies, or hereditary syndromes. She was a nonsmoker, and no psychosocial or behavioral factors likely to influence orthodontic treatment were identified.

Her dental history revealed previous extraction of the maxillary right second molar and mandibular left first molar because of extensive dental caries. The patient had not received previous orthodontic treatment.

### 2.2. Clinical Findings

**Figure 1:**
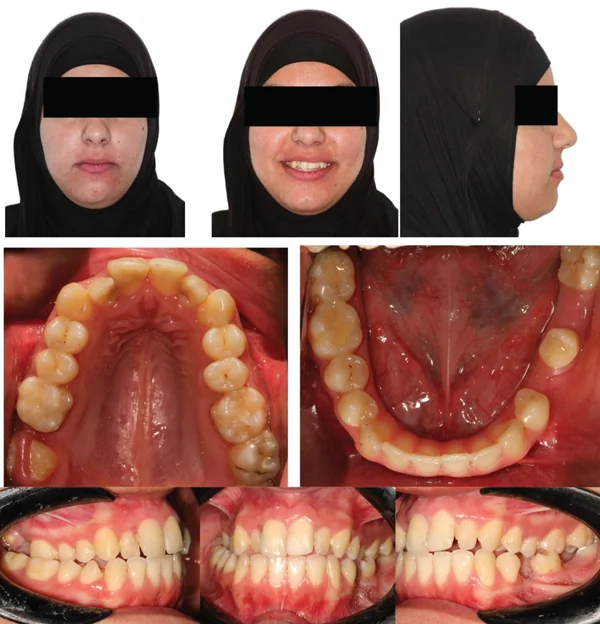
Initial clinical photographs.

Extraoral examination demonstrated mild chin deviation to the right, increased vertical facial proportions, and a straight facial profile. The maxillary dental midline was coincident with the facial midline. Lip competence was present at rest, and appropriate maxillary incisor display was observed during smiling (Figure 1).

Intraoral examination revealed a constricted maxillary arch with a unilateral posterior crossbite on the right side and a crossbite involving the maxillary right lateral incisor. The patient exhibited reduced overjet and overbite, a Class I molar relationship on the right side, a one‐quarter‐unit Class II canine relationship on the right side, and a one‐half‐unit Class II canine relationship on the left side. The maxillary and mandibular dental midlines were coincident with the facial midline. Rotation of the mandibular left second premolar was also observed (Figure 1).

No swelling, tenderness, periodontal pocketing, or signs of acute infection were associated with the impacted mandibular left second molar.

The chronological sequence of the diagnostic procedures and treatment is summarized in Table 1.

**Table 1:** Timeline.

Time	Clinical event
Initial presentation	An 18‐year‐old female presented seeking orthodontic treatment because of dissatisfaction with her smile aesthetics.
Initial clinical assessment	Extraoral and intraoral examinations were performed. Panoramic radiography, lateral cephalometric radiography, and CBCT demonstrated a deeply impacted mandibular left second molar with severe root dilaceration, intimate proximity to the inferior alveolar canal, and a pericoronal radiolucent lesion radiographically consistent with a dentigerous cyst (Figures 1 and 2).
Treatment planning	Following interdisciplinary consultation with oral and maxillofacial surgeons, orthodontic traction was selected as a conservative alternative to surgical extraction because of the increased risk of inferior alveolar nerve injury.
Months 0–12	Maxillary expansion, alignment, and leveling of both dental arches; space creation for the impacted mandibular left second molar; and uprighting of the mandibular left third molar were completed.
Month 12	Surgical exposure of the impacted mandibular left second molar, marsupialization of the pericoronal lesion, bonding of a gold chain, and placement of a mandibular temporary anchorage device (TAD). Orthodontic traction was initiated (Figure 3).
Months 12–20	Progressive orthodontic traction using skeletal anchorage resulted in gradual uprighting and extrusion of the impacted mandibular left second molar (Figures 4 and 5).
Months 20–26	A individualized auxiliary levering arm and additional skeletal anchorage were incorporated to modify the force vector and facilitate further uprighting and eruption (Figures 4, 5, and 6).
Months 26–30	The mandibular left second molar erupted into functional occlusion, followed by finishing, detailing, and removal of the fixed orthodontic appliance (Figures 4 and 7).
Six‐month follow‐up	Stable occlusion, positive pulp vitality, satisfactory periodontal health, and no clinical evidence of neurosensory complications or relapse were observed.

## 3. Diagnostic Assessment

Clinical examination was supplemented with panoramic radiography, lateral cephalometric analysis, and cone‐beam computed tomography (CBCT).

**Figure 2:**
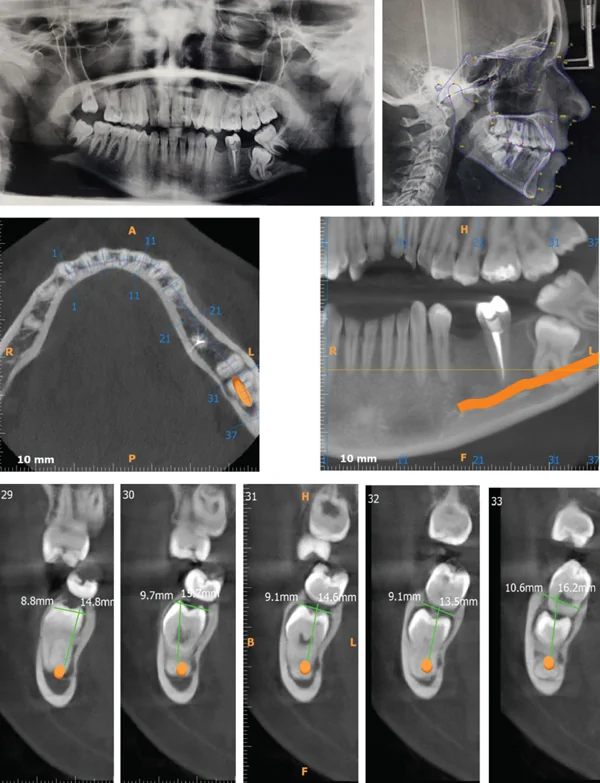
Initial panoramic radiograph, cephalogram, and CBCT.

Panoramic radiography demonstrated a deeply impacted mandibular left second molar with severe root dilaceration. The mandibular left first molar and the maxillary right second molar were absent because of previous extraction due to extensive dental caries (Figures 2).

Cephalometric analysis demonstrated a skeletal Class I relationship with increased vertical facial proportions (MMPA = 34^°^). The maxillary and mandibular incisors exhibited normal inclination relative to their respective skeletal bases.

Because maxillary constriction may be associated with altered respiratory patterns, the patient was clinically evaluated for possible contributing etiological factors. No clinical history or examination findings suggestive of chronic mouth breathing, adenoid hypertrophy, or other upper airway obstruction were identified. As no specific etiological factor could be identified clinically, the maxillary constriction was considered to be multifactorial in origin.

CBCT confirmed the intimate relationship between the roots of the impacted mandibular left second molar and the inferior alveolar canal. The roots exhibited marked dilaceration, and a well‐defined pericoronal radiolucent lesion surrounding the crown was observed, radiographically consistent with a dentigerous cyst (Figure 2) [10].

The principal diagnostic challenge was determining whether the impacted mandibular left second molar could be preserved despite its intimate relationship with the inferior alveolar canal. Immediate surgical extraction was considered to carry a substantial risk of inferior alveolar nerve injury. Consequently, CBCT evaluation and interdisciplinary consultation with oral and maxillofacial surgeons were essential in determining the most appropriate treatment strategy.

The sequence of diagnostic evaluation, differential diagnosis, and treatment planning is summarized in Table 1.

Ankylosis was considered in the differential diagnosis because of the severe impaction and absence of eruption. However, gentle intraoperative luxation confirmed physiologic tooth mobility, effectively excluding ankylosis.

## 4. Final Diagnosis

The patient′s final diagnosis are as follows:

• Skeletal Class I malocclusion with increased vertical facial proportions.
• Maxillary transverse deficiency with a unilateral posterior crossbite.
• Deeply impacted mandibular left second molar with severe root dilaceration.
• Pericoronal radiolucent lesion surrounding the crown of the impacted mandibular left second molar, radiographically consistent with a dentigerous cyst.
• Intimate proximity of the impacted mandibular left second molar to the inferior alveolar canal.
• Previous extraction of the maxillary right second molar because of extensive dental caries.
• Previous extraction of the mandibular left first molar because of extensive dental caries.

## 5. Treatment Objectives

The treatment objectives were to

• Preserve the impacted mandibular left second molar whenever feasible while minimizing the risk of injury to the inferior alveolar nerve.
• Create sufficient space to facilitate orthodontic traction of the impacted tooth.
• Expand the maxillary arch and correct the transverse discrepancy.
• Correct the anterior and posterior crossbites.
• Establish normal overjet and overbite.
• Achieve a stable functional occlusion with appropriate canine and molar relationships.
• Improve smile esthetics while maintaining long‐term dental and periodontal health.

## 6. Treatment Alternatives

Following comprehensive clinical and radiographic evaluation, an interdisciplinary discussion was conducted between the orthodontic and oral and maxillofacial surgery teams to determine the most appropriate treatment approach.

Several treatment options were considered and discussed with the patient:

1. Surgical extraction of the impacted mandibular left second molar under general anesthesia. However, this option was associated with a considerable risk of inferior alveolar nerve injury because CBCT demonstrated intimate contact between the dilacerated roots and the inferior alveolar canal.
2. Extraction of the impacted mandibular left second molar followed by implant‐supported rehabilitation of the missing mandibular left first molar. Immediate implant placement was not feasible because of the position of the impacted tooth and the limited amount of available bone.
3. Retention of the impacted mandibular left second molar with prosthetic replacement of the missing mandibular left first molar using a fixed dental prosthesis. This option was considered less favorable because the retained impacted tooth could predispose the patient to future pathological changes and potentially compromise the adjacent mandibular third molar.
4. Orthodontic traction to reposition the impacted mandibular left second molar into functional occlusion. If adequate orthodontic movement could not be achieved or if complications developed during treatment, surgical extraction would be reconsidered after increasing the distance between the tooth and the inferior alveolar canal.
5. Extraction of the impacted mandibular left second molar followed by orthodontic mesialization of the mandibular left third molar to replace the missing mandibular left first molar.
6. Coronectomy to reduce the risk of inferior alveolar nerve injury while retaining the roots.
7. Periodic clinical and radiographic observation without active intervention.

After discussing the advantages, disadvantages, expected treatment duration, possible complications, and long‐term prognosis of each treatment option, the patient elected to proceed with orthodontic traction in an attempt to preserve the natural tooth. Considering the patient′s age, excellent motivation, absence of ankylosis, and the increased surgical risk associated with extraction, conservative orthodontic management was considered the most appropriate treatment strategy [9, 11, 16, 17].

## 7. Therapeutic Intervention

The chronological course of treatment is summarized in Table 1.

Comprehensive orthodontic treatment was initiated with transverse expansion of the maxillary arch using a quad‐helix appliance to correct the maxillary constriction. Fixed orthodontic appliances with a 0.022 × 0.028 − inch MBT prescription were subsequently bonded in both arches. Alignment and leveling were achieved using a sequence of nickel–titanium archwires followed by stainless‐steel archwires until a passive 0.019 × 0.025 − inch stainless‐steel working archwire was reached.

To facilitate eruption of the impacted mandibular left second molar, adequate space was first created within the mandibular arch. A nickel–titanium open‐coil spring was used to distalize the mandibular left second premolar while simultaneously promoting mesial uprighting of the mandibular left third molar, thereby reducing mechanical interference with the eruption pathway of the impacted tooth.

Following approximately 12 months of orthodontic preparation, a 1.4 × 6 mm TAD was inserted between the mandibular left first and second premolars to provide absolute anchorage during the initial phase of orthodontic traction.

**Figure 3:**
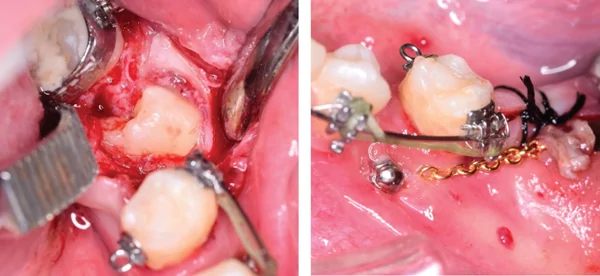
Surgical exposure and gold chain attachment.

A full‐thickness mucoperiosteal flap was reflected to expose the impacted mandibular left second molar. Marsupialization of the associated pericoronal lesion was performed to decompress the lesion and facilitate tooth eruption. Because the lesion was managed conservatively, histopathological examination was not performed. Gentle luxation confirmed that the tooth was not ankylosed. A gold chain was bonded to the occlusal surface of the impacted tooth and immediately connected to the mandibular TAD using elastic power thread (Figure 3).

Light, continuous orthodontic forces of approximately 80–100 g were applied to initiate traction. The force magnitude was deliberately maintained within a biologically acceptable range to promote gradual tooth movement while minimizing the risk of root resorption, periodontal damage, and excessive stress on the surrounding bone. The elastic power thread was assessed and reactivated at approximately 4‐week intervals throughout treatment [2, 12, 16, 17].

Because the initial position of the tooth was nearly horizontal and the crown was located beneath the distal aspect of the mandibular left third molar, direct vertical extrusion at the beginning of treatment was considered biomechanically unfavorable. Therefore, the initial force vector was directed occlusomesially to disengage the crown from beneath the third molar and achieve gradual uprighting before vertical eruption.

This biomechanical approach was selected because direct vertical extrusion of the deeply impacted mandibular left second molar would have generated unfavorable force vectors and increased the likelihood of interference with the adjacent mandibular left third molar. Initial occlusomesial traction allowed progressive uprighting of the tooth, reduced mechanical obstruction from the third molar, and facilitated subsequent vertical eruption. The use of TADs provided absolute anchorage, minimizing undesirable reciprocal tooth movement while allowing precise control of the magnitude and direction of orthodontic forces throughout treatment [2, 9, 12, 16, 17].

**Figure 4:**
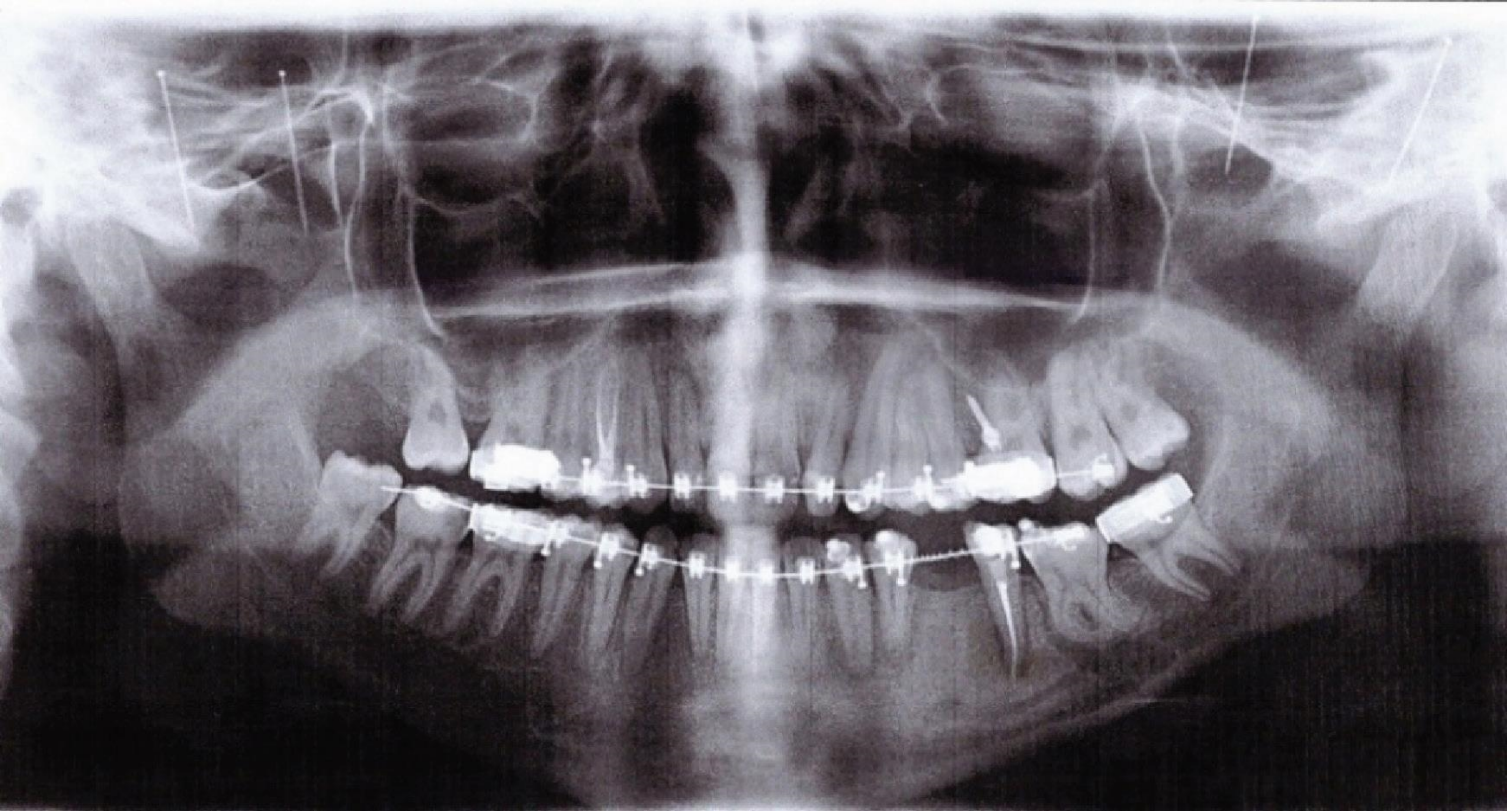
Sequential panoramic radiographs.

**Figure 5:**
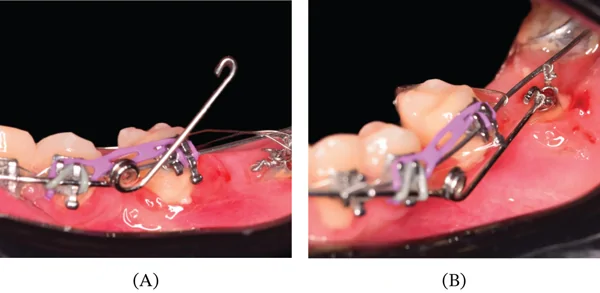
Individualized TMA auxiliary levering arm: (A) deactivated and (B) activated.

Serial panoramic radiographs demonstrated progressive movement of the impacted mandibular left second molar throughout treatment (Figure 4). As the inclination of the tooth improved, a customized auxiliary levering arm fabricated from 0.7‐mm titanium‐molybdenum alloy (TMA) wire was incorporated to optimize the force system (Figure 5). The auxiliary increased the distance between the point of force application and the center of resistance of the impacted tooth, thereby improving rotational control and facilitating controlled uprighting.

**Figure 6:**
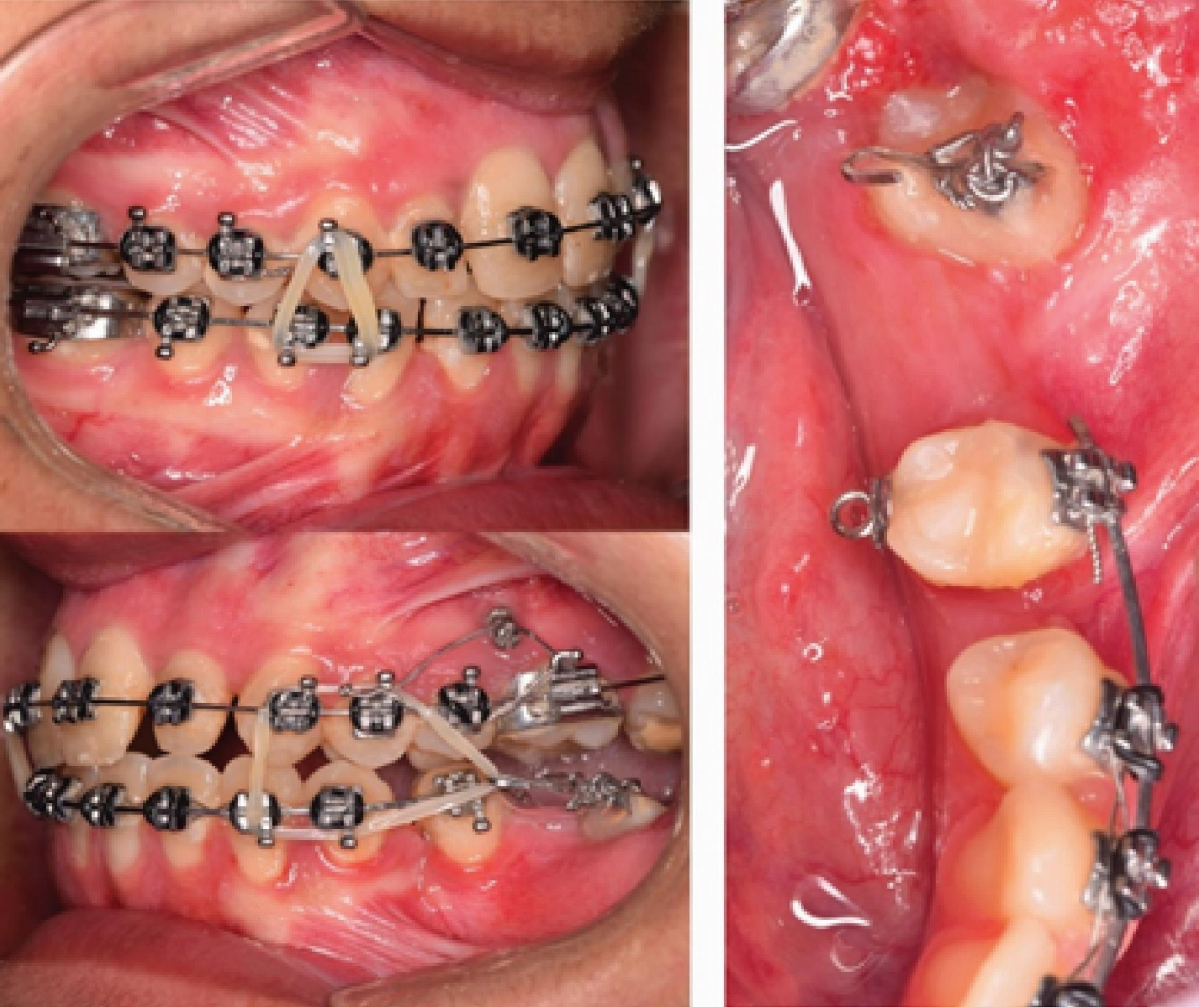
Additional maxillary TAD used as indirect anchorage for traction of the impacted mandibular left second molar using a 3/16‐inch elastic attached to an S‐shaped hook.

During the later stages of treatment, an additional TAD was inserted between the maxillary left second premolar and first molar and used as indirect anchorage. A 3/16‐inch elastic was attached from the maxillary anchorage unit to an S‐shaped hook incorporated into the auxiliary system to redirect the force vector toward a more vertical direction, thereby facilitating continued eruption into the dental arch while minimizing undesirable reciprocal tooth movement (Figure 6) [12, 16, 17].

The patient demonstrated excellent compliance throughout treatment by attending scheduled appointments regularly, maintaining satisfactory oral hygiene, and adhering to all treatment instructions. This high level of cooperation contributed significantly to the successful treatment outcome.

## 8. Follow‐Up and Outcomes

The total active treatment duration was 30 months.

**Figure 7:**
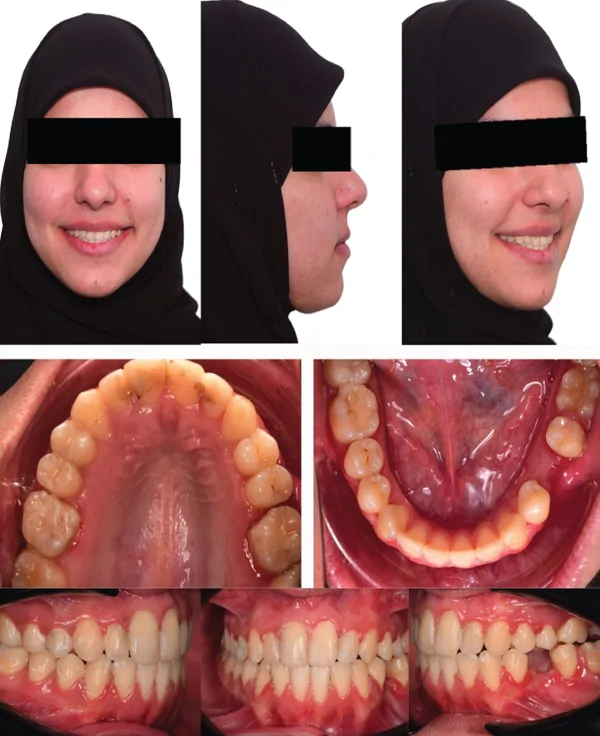
Final clinical photographs.

Posttreatment extraoral photographs demonstrated an improved smile with appropriate incisor display, preservation of the straight facial profile, and satisfactory facial symmetry (Figure 7).

Intraoral examination demonstrated successful eruption of the mandibular left second molar into functional occlusion. A stable occlusal relationship was achieved, including a Class I molar relationship on the right side, bilateral Class I canine relationships, correction of the anterior and posterior crossbites, normal overjet and overbite, and satisfactory alignment of both dental arches (Figure 7).

Adequate space was preserved for future implant placement in the mandibular left first molar region. Endodontic retreatment of the mandibular left second premolar was successfully completed before prosthetic rehabilitation.

Serial panoramic radiographs obtained throughout treatment demonstrated progressive uprighting and eruption of the impacted mandibular second molar until functional occlusion was achieved (Figure 4). Because postoperative CBCT was not performed, the final relationship between the tooth roots and the inferior alveolar canal was assessed using panoramic radiography together with clinical examination.

Pulp vitality testing demonstrated a positive response of the mandibular left second molar, indicating preservation of pulpal vitality following orthodontic traction.

No intraoperative or postoperative complications occurred during treatment. Throughout treatment and during the 6‐month follow‐up period, the patient remained free of pain, infection, pulp necrosis, periodontal complications, or clinical evidence of inferior alveolar nerve dysfunction, including paresthesia or numbness of the lower lip. Occlusal stability was maintained, and no clinical evidence of relapse was observed during the follow‐up period.

The patient expressed a high level of satisfaction with both the functional and esthetic outcomes and preferred preservation of the natural tooth over extraction and prosthetic replacement.

## 9. Discussion

Deep impaction of a mandibular second molar associated with severe root dilaceration and intimate proximity to the inferior alveolar canal represents an uncommon and clinically challenging presentation. Successful management of such cases requires careful diagnosis, interdisciplinary treatment planning, and individualized biomechanics because both the complex root morphology and the risk of inferior alveolar nerve injury may complicate treatment. Recent radiographic and clinical studies have emphasized that CBCT is indispensable for evaluating root morphology, the spatial relationship with the inferior alveolar canal, and associated pathological lesions, thereby facilitating more accurate treatment planning and reducing surgical risk [6, 8, 9]. The recent systematic review by Barone et al. [9] further highlighted the importance of individualized treatment planning based on three‐dimensional anatomical assessment rather than relying solely on conventional radiographs. In the present case, these challenges were further compounded by the presence of a pericoronal radiolucent lesion radiographically consistent with a dentigerous cyst and the nearly horizontal orientation of the impacted tooth.

Management of impacted mandibular second molars remains controversial, and treatment should be individualized according to patient age, root development, tooth position, associated pathology, and the relationship of the tooth to adjacent anatomical structures. Reported treatment options include observation, surgical extraction, coronectomy, spontaneous eruption following removal of the third molar, and orthodontic uprighting or traction [7–9, 11–17]. A recent systematic review and meta‐analysis concluded that no single treatment modality is universally superior and recommended individualized treatment planning based on the severity of impaction, root morphology, and anatomical considerations [9]. Similarly, Chen et al. [8] reported that treatment selection should be guided by patient age, root development, impaction severity, and the proximity of the tooth to adjacent anatomical structures, particularly the inferior alveolar canal. In the present case, consultation with oral and maxillofacial surgeons indicated that immediate surgical extraction carried a considerable risk of inferior alveolar nerve injury because of the intimate relationship between the dilacerated roots and the inferior alveolar canal. Consequently, conservative orthodontic traction was selected as the preferred treatment approach.

Preservation of the natural tooth was considered preferable because successful eruption would preserve the patient′s natural dentition, eliminate or postpone the need for implant‐supported rehabilitation in a young patient, and avoid the potential neurosensory risks associated with immediate surgical extraction. Furthermore, this conservative strategy preserved future treatment options, as surgical extraction would still have remained feasible if orthodontic traction had proved unsuccessful. This treatment philosophy is consistent with the current trend toward minimally invasive management whenever preservation of the natural dentition is considered biologically and clinically feasible [8, 9].

The biomechanics employed in this case differed from those commonly described in the literature. Most published reports describe partially impacted or mesially inclined mandibular second molars that can often be uprighted using relatively simple mechanics [12–17]. In contrast, the present case involved a deeply impacted tooth with a nearly horizontal orientation, severe root dilaceration, and obstruction by the adjacent mandibular third molar. Direct vertical traction at the beginning of treatment would have generated an unfavorable force vector and increased the likelihood of interference with the third molar. Therefore, the initial force vector was directed occlusomesially to disengage the impacted tooth, facilitate gradual uprighting, and create a more favorable path for subsequent vertical eruption. This staged biomechanical approach is consistent with recent reports emphasizing that force direction should be modified throughout treatment according to changes in tooth position rather than relying on a single line of traction from the outset [16, 17]. Lorente et al. [16] also demonstrated that modifying the force vector during treatment improves the efficiency of molar uprighting while maintaining stable anchorage. More recently, Yuan et al. [17] confirmed the effectiveness of skeletal anchorage in controlling complex force systems for impacted mandibular second molars.

TADs provided absolute anchorage throughout treatment, allowing controlled force application without undesirable reciprocal tooth movement. In addition, the customized auxiliary levering arm improved the moment‐to‐force ratio and enabled progressive modification of the force direction as the position of the impacted tooth changed. This biomechanical approach allowed controlled orthodontic movement while minimizing excessive loading of the periodontal tissues and surrounding bone. Recent prospective clinical studies by Lorente et al. [16] and Yuan et al. [17] demonstrated that skeletal anchorage provides predictable control of force direction while minimizing undesirable reciprocal tooth movement and maintaining favorable periodontal outcomes. The present case supports these observations, as progressive modification of the force vector using two TADs enabled controlled eruption despite the exceptionally unfavorable initial position of the tooth. Serial panoramic radiographs demonstrated progressive eruption of the impacted mandibular left second molar throughout treatment, ultimately resulting in successful positioning within the dental arch.

The etiology of root dilaceration is considered multifactorial. Previous studies have implicated developmental disturbances, trauma, ectopic positioning of the tooth germ, space deficiency, odontogenic lesions, and mechanical interference from adjacent anatomical structures as possible contributing factors [10, 18–26]. Enache et al. [10] proposed that prolonged pressure exerted by a dentigerous cyst may interfere with Hertwig′s epithelial root sheath during root development, potentially contributing to root dilaceration and abnormal root morphology. In the present case, although histopathological confirmation of a dentigerous cyst was not available, the pericoronal radiolucent lesion radiographically consistent with a dentigerous cyst may have contributed to both tooth displacement and the severe root dilaceration observed. In addition, the developing mandibular third molar may have imposed further mechanical constraints that increased the severity of the impaction and root morphology.

Early treatment of impacted mandibular second molars is generally recommended, particularly between 11 and 14 years of age, when root development remains incomplete and orthodontic movement is considered more favorable [7, 8]. However, recent systematic evidence indicates that successful outcomes remain achievable in carefully selected older patients when modern biomechanical strategies and skeletal anchorage are employed [9, 11, 17]. Nevertheless, the successful outcome achieved in this 18‐year‐old patient demonstrates that age alone should not be regarded as a contraindication to orthodontic traction. Careful diagnosis, appropriate case selection, skeletal anchorage, and individualized biomechanics may still permit successful eruption even after complete root formation.

The principal strength of this report lies in the successful management of a rare combination of deep impaction, severe root dilaceration, horizontal orientation, a pericoronal radiolucent lesion radiographically consistent with a dentigerous cyst, and intimate proximity to the inferior alveolar canal using conservative orthodontic treatment. Recent prospective studies have reported high success rates for skeletal anchorage‐assisted uprighting of impacted mandibular second molars; however, most involved mesially inclined or partially impacted teeth rather than deeply impacted teeth with severe root dilaceration and intimate proximity to the inferior alveolar canal [16, 17]. This case therefore expands the limited body of evidence supporting orthodontic traction as a feasible alternative to surgical extraction in carefully selected high‐risk patients.

Several limitations should be acknowledged. Histopathological examination of the pericoronal lesion was not performed following marsupialization; therefore, the diagnosis of a dentigerous cyst was based on the clinical and radiographic findings rather than histopathological confirmation. In addition, postoperative CBCT was not obtained because routine three‐dimensional imaging was not considered clinically justified following successful treatment in the absence of neurosensory symptoms. Consequently, assessment of the final relationship between the tooth roots and the inferior alveolar canal relied on panoramic radiography in conjunction with clinical examination. Finally, the follow‐up period was limited to 6 months after appliance removal, and longer‐term observation would be valuable to evaluate the stability of the treatment outcome, periodontal health, and long‐term pulpal status.

From a clinical perspective, this case highlights the importance of comprehensive CBCT‐based diagnosis, interdisciplinary collaboration, individualized biomechanical planning, and the use of skeletal anchorage in the management of complex impacted mandibular second molars. This approach is consistent with contemporary systematic reviews and recent clinical studies supporting individualized treatment planning, CBCT‐guided diagnosis, and skeletal anchorage‐assisted biomechanics as predictable strategies for preserving impacted mandibular second molars whenever feasible [8, 9, 16, 17]. In carefully selected patients, conservative orthodontic traction should be considered a predictable and biologically favorable alternative to surgical extraction when preservation of the natural dentition is feasible and the risk of inferior alveolar nerve injury is substantial.

## 10. Conclusion

The management of deeply impacted mandibular second molars associated with severe root dilaceration and intimate proximity to the inferior alveolar canal presents a significant clinical challenge. This case demonstrates that successful preservation of the impacted tooth can be achieved through comprehensive CBCT‐based diagnosis, interdisciplinary collaboration, individualized biomechanical planning, and controlled orthodontic traction supported by TADs. Although such cases are uncommon, conservative orthodontic management should be considered a viable alternative to surgical extraction in carefully selected patients, particularly when the risk of inferior alveolar nerve injury is substantial. Additional clinical reports and longer‐term follow‐up studies are needed to further evaluate the predictability, long‐term stability, and clinical outcomes of this treatment approach.

**Nomenclature**
CBCT: cone‐beam computed tomography
IAC: inferior alveolar canal
IAN: inferior alveolar nerve
MMPA: maxillary–mandibular plane angle
OPG: orthopantomogram
TADs: temporary anchorage devices

## Author Contributions

**Mutaz Arman:** original draft preparation (lead), conceptualization (lead), data curation (equal), formal analysis (equal), investigation (lead), project administration (equal), software (equal), supervision (equal), validation (equal), visualization (equal), review and editing (lead). **George Dahabreh:** methodology (lead), resources (lead), data curation (equal), formal analysis (equal), funding acquisition (lead), investigation (supporting), project administration (equal), software (equal), supervision (equal), validation (equal), visualization (equal), review and editing (supporting).

## Funding

No funding was received for this manuscript.

## Disclosure

All authors have read and approved the final version of the manuscript. Mutaz Arman had full access to all of the data in this study and takes complete responsibility for the integrity of the data and the accuracy of the data analysis.

## Ethics Statement

This study is a case report and does not involve experimental research. Ethical approval was not required for this case report according to the policies of the Faculty of Dentistry, Al‐Quds University, as it describes the management of a single clinical case. The report was prepared in accordance with the principles of the Declaration of Helsinki.

## Consent

Written informed consent was obtained from the patient for treatment and publication of the clinical information, radiographs, and photographs included in this manuscript. Moreover, a second consent to publish “Patient Consent Form by Wiley” was obtained retrospectively from the patient during the preparation of the scientific article. A copy of the written consent is available for review by the editor upon request.

## Conflicts of Interest

The authors declare no conflicts of interest.

## Patient Perspective

The patient expressed a strong preference for preserving the impacted mandibular second molar and avoiding surgical extraction because of the potential risk of inferior alveolar nerve injury. She understood that orthodontic traction would require prolonged treatment and multiple surgical and orthodontic procedures but remained highly motivated and compliant throughout the treatment period. At the completion of treatment, the patient reported satisfaction with both the functional and esthetic outcomes. She particularly appreciated the successful eruption and preservation of her natural tooth, which eliminated the need for immediate surgical extraction and prosthetic replacement.

## Supporting information


**Supporting Information** Additional supporting information can be found online in the Supporting Information section. 

## Data Availability

The data that support the findings of this study are available from the corresponding author upon reasonable request.
